# Metabolic Signatures of *Acinetobacter baumannii* and *Klebsiella pneumoniae* Infections in Acute-on-chronic Liver Failure

**DOI:** 10.1016/j.jcmgh.2025.101707

**Published:** 2025-12-12

**Authors:** Camilla Cadoli, Sania Arif, Wibke Ballhorn, Angela Brieger, Maximilian Joseph Brol, Florence Castelli, Hans-Peter Erasmus, Julia Fischer, Robert Gurke, Lisa Hahnefeld, Christophe Junot, Nico Kraus, Cristina Ortiz, Robert Schierwagen, Sara Garcia Torres, Frank Erhard Uschner, Volker Müller, Jonel Trebicka, Christoph Welsch, Volkhard A.J. Kempf

**Affiliations:** 1 Institute of Medical Microbiology and Infection Control, Goethe University Frankfurt, University Hospital, Frankfurt am Main, Germany; 2 Department of Medicine B (Gastroenterology, Hepatology, Endocrinology, Clinical Infectiology), University Hospital Muenster, Muenster, Germany; 3 Medical Clinic 1, Biomedical Research Laboratory, Goethe University Frankfurt, University Hospital, Frankfurt am Main, Germany; 4 Département Médicaments et Technologies pour la Santé (MTS), Université Paris-Saclay, CEA, INRAE, Gif-sur-Yvette, France; 5 MetaboHUB, Gif-sur-Yvette, France; 6 Molecular Hepatology and Inflammation Research, Goethe University Frankfurt, University Hospital, Frankfurt am Main, Germany; 7 Faculty of Medicine, Institute of Clinical Pharmacology, Frankfurt am Main, Germany; 8 Fraunhofer Institute for Translational Medicine and Pharmacology ITMP, Frankfurt am Main, Germany; 9 Fraunhofer Cluster of Excellence Immune-mediated Diseases CIMD, Frankfurt am Main, Germany; 10 Institute of Molecular Biosciences, Goethe University Frankfurt, Frankfurt am Main, Germany; 11 European Foundation for the Study of Chronic Liver Failure - EF CLIF, Barcelona, Spain

**Keywords:** Infection-associated Metabolites, Liver Disease, Metabolomic Profiling, Potential Biomarkers

## Abstract

**Background & Aims:**

Acute-on-chronic liver failure (ACLF) is a life-threatening syndrome of acute hepatic decompensation (AD) that leads to multiorgan failure and high mortality. Bacterial infections are often implicated in ACLF pathogenesis; however, their underlying molecular mechanisms remain poorly understood. This study employed a combined in vitro-ex vivo metabolomics approach to investigate infection-associated metabolic alterations relevant to ACLF.

**Methods:**

Gut (Caco-2) cells were infected with *Acinetobacter baumannii* and *Klebsiella pneumoniae* strains. Metabolite profiling was conducted on cell culture supernatants, and selected metabolites were tested for hepatotoxicity in vitro using liver (HepG2) cells. Metabolomic analysis of sera from 2 independent patient cohorts (AD and ACLF) was conducted to validate in vitro findings and to assess their clinical relevance.

**Results:**

Distinct metabolic signatures were identified in *Abaumannii* (19 metabolites) and *Kpneumoniae* (15 metabolites)-infected Caco-2 cells. Four key metabolites from each bacterial species were prioritized for further experiments: α-ketoglutarate, indoleacetic acid, p-coumaric acid, uridine (*Abaumannii*), desthiobiotin, N8-acetylspermidine, N-acetylglutamine, and β-pinene (*Kpneumoniae*). Hepatotoxicity was demonstrated in liver (HepG2) cells exposed to Caco-2 infected cell-derived supernatants, infection-associated metabolites, and metabolite mixtures (in all conditions, *P* < .0001). Increased levels of α-ketoglutarate (*P* = .0002), N-acetylglutamine (*P* = .0153), indoleacetic acid (*P* < .05), and N8-acetylspermidine (*P* < .01) have been confirmed in the sera of patients with AD and ACLF.

**Conclusions:**

Our findings suggest that metabolites associated with bacterial infections and hepatotoxic potential are significantly elevated in patients with AD and ACLF. These compounds may contribute to disease-related metabolic disturbances, representing promising candidates as early diagnostic biomarkers and targeted therapeutic strategies for ACLF.

## Graphical abstract

**Figure:**
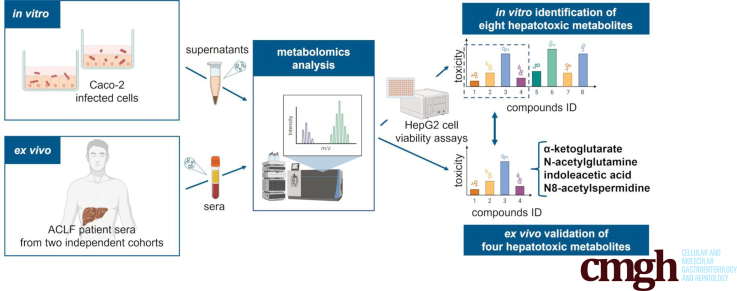

**Summary**

Bacterial infections contribute to acute-on-chronic liver failure onset. In vitro models reveal that metabolites from *Acinetobacter baumannii* and *Klebsiella pneumoniae* infection induce specific hepatotoxic compounds, which were subsequently detected in sera of patients with acute hepatic decompensation and sera from patients with acute-on-chronic liver failure.

**What You Need to Know**

**Background**

Bacterial infections often trigger acute-on-chronic liver failure, yet mechanisms remain unclear. This study uses metabolomics and in vitro–ex vivo models to identify hepatotoxic infection-associated metabolites.

**Impact**

Distinct metabolites from *Acinetobacter baumannii* and *Klebsiella pneumoniae* infections induce hepatocyte injury and are elevated in sera from patients with acute decompensation and acute-on-chronic liver failure, representing candidate biomarkers and targeted therapeutic strategies.

**Future Directions**

These findings pave the way for the development of biomarkers, targeted therapies, and modulation of the gut-liver axis to prevent infection-associated liver injury in patients with acute decompensation and acute-on-chronic liver failure.

Acute-on-chronic liver failure (ACLF) is a clinical syndrome defined by acute hepatic decompensation (AD) in individuals with advanced chronic liver disease, leading to multi-organ failure and high short-term mortality, often within weeks.^1^ Infections are and alcohol abuse are common triggers, with bacterial infections accounting for 35% of cases globally.^2^ Bacteria and bacterial metabolite translocation into the liver activate inflammatory cascades, worsen portal hypertension, and accelerate liver injury.^3^^,^^4^ Despite its clinical significance, ACLF pathogenesis remains insufficiently characterized. Emerging evidence highlights the gut-liver axis as a key driver, where gut dysbiosis, an imbalance in microbial composition, and compromised barrier integrity, enables microbial products to enter portal circulation, fueling hepatic inflammation and organ dysfunction.^3^, ^4^, ^5^ Frequent hospitalization and the use of broad-spectrum antibiotics in patients with ACLF further disrupt microbial homeostasis, promoting pathological overgrowth of antibiotic-resistant bacteria in the gut.^6^ *Klebsiella pneumoniae* and *Acinetobacter baumannii* have been prioritized by the World Health Organization (WHO) as urgent health threats due to their carbapenem resistance and increasing clinical prevalence.^7^ *K* *pneumoniae*, a dominant gut colonizer, is linked to spontaneous bacterial peritonitis and bloodstream infections, common ACLF precipitants.^8^ *A* *baumannii* causes severe nosocomial infections in immunocompromised hosts, including those with liver disease.^7^^,^^9^ In ACLF, colonization by these pathogens can rapidly escalate to invasive infection and organ failure.^7^^,^^9^ Subsequent metabolic dysregulation affects multiple organ systems, involving alterations in amino acids, lipids, bile acids, and microbial-derived compounds, with bacterial infections driving shifts in energy metabolism and disease severity.^10^^,^^11^ Metabolites, byproducts of microbial and host metabolism, can both sustain homeostasis and disrupt metabolic balance.^10^^,^^12^ To reflect microbial dynamics in patients with ACLF, this study investigates the metabolic impact of *A* *baumannii* and *K* *pneumoniae* on the disease using laboratory reference strains,^13^^,^^14^ patient-derived multidrug-resistant (carbapenem-resistant), and highly virulent clinical strains.^15^^,^^16^ This study leverages metabolomics, a comprehensive analysis of metabolites within biological systems, to infection models and patient datasets. This strategy aims to identify profile metabolic alterations and infection-associated metabolites in ACLF, providing insights into the interplay between bacterial infections and host metabolic imbalance, paving the way for innovative therapeutic strategies.

## Results

### Infections of Caco-2 cells With *Abaumannii* or *Kpneumoniae*

**Figure 1:**
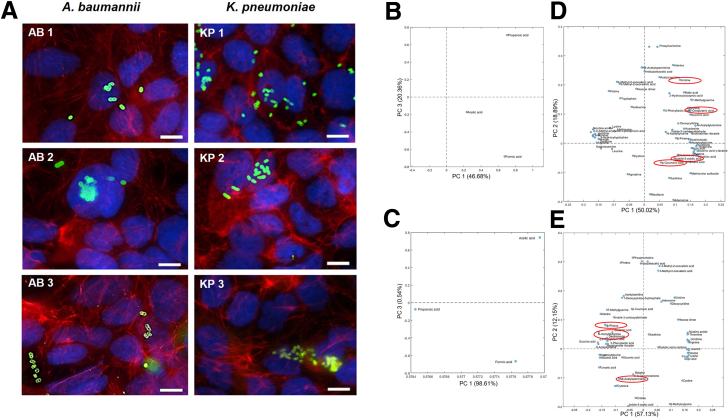
**Caco-2 cells infected 6 hours with *Abaumannii* or *Kpneumoniae*.** (*A*) Double-fluorescence microscopy of Caco-2 cells following a 6-hour infection with *Abaumannii* (AB) or *Kpneumoniae* (KP) strains. Extracellular bacteria are stained in *green* (Alexa-488), intracellular bacteria in *orange* (Alexa-647). The cytoskeleton is stained in *red* (TRITC-phalloidin), DNA and nuclei in *blue* (DAPI). Scale bar: 10 μm. Fluorescence microscopy analysis was performed with the microscope Zeiss Axio Imager 2 (Zeiss) equipped with a Spot RT3 camera (Diagnostic Instruments Inc) and operated by VisiView V.2.0.5 (Visitron Systems). Images were acquired with 63× objective. (*B, C*) Targeted metabolomics of analysis of Caco-2 cell-culture supernatants collected after 6-hour infection with *Abaumannii* (*B*) or *Kpneumoniae* (*C*) strains. Controls included supernatants from uninfected Caco-2 cells and bacterial cultures incubated without host cells under identical conditions. Metabolomics profiling was performed on 2 biological replicates per condition, from 2 independent experiments, each consisting of 3 replicates pooled at the supernatant level. Loading plots from PCA models calculated on the relative concentrations of SCFAs in the reduced datasets are depicted. These plots reveal distinct metabolic shifts across experimental conditions, highlighting key metabolites contributing to group separation in PCA space. (*D, E*) Untargeted metabolomics analysis of Caco-2 cell-culture supernatants collected after 6-hour infection with *Abaumannii* (*D*) or *Kpneumoniae* (*E*) strains. Controls included 6-hour incubated supernatants from uninfected Caco-2 cells and bacterial cultures incubated without host cells under identical conditions. Metabolomics profiling was performed on 2 biological replicates per condition, from 2 independent experiments, each consisting of 3 replicates pooled at the supernatant level. Loading plots from PCA models were generated based on the relative concentrations of metabolites annotated on level 1 and level 2a in the reduced datasets. These plots reveal distinct metabolic shifts across experimental conditions, highlighting key metabolites contributing to group separation in PCA space.

ACLF is often worsened by bacterial infections with Gram-negative bacteria due to compromised intestinal barrier integrity, allowing microbial translocation and further liver damage.^3^^,^^17^ To emulate this in vivo response, we employed gut cells (Caco-2) with various *A* *baumannii* and *K* *pneumoniae* strains (Table 1; see Materials and Methods). A 6-hour infection window was selected to preserve cell integrity, avoid bacterial overgrowth, and ensure consistency across all bacterial strains. Infections were conducted at MOI 100 (*A baumannii*) and MOI 1 (*K* *pneumoniae*) and analyzed via double immunofluorescence (Figure 1*A*). Microscopy showed no major differences in cellular phenotypes across infections with reference, multidrug-resistant (MDR), or highly virulent strains, supporting the physiological relevance of the infection conditions.

**Table 1:** Bacterial Strains Used in This Study

Strains	Source of isolation	Year of isolation	Origin	Resistance genes
*Acinetobacter baumannii*				
AB 1: wild-type *Abaumannii*^13^	Urine	1948	ATCC 19606^T^	*sul2*
AB 2: MDR	Nose swab	2011	Patient isolate (UHF^a^)	*blaOXA-23*
AB 3: MDR and highly virulent	Rectal swab	2015	Patient isolate (UHF^a^)	*blaOXA-23*^b^^,^^15^
*Klebsiella pneumoniae*				
KP 1: wild-type *Kpneumoniae*^14^	Urine	1994	ATCC 700603	*blaSHV-18*
KP 2: MDR	Throat swab	2019	Patient isolate (UHF^a^)	*blaOXA-48*
KP 3: MDR and highly virulent	Throat swab	2013	Patient isolate (UHF^a^)	*blaOXA-48*^b^^,^^16^

AB, *Acinetobacter baumannii*; KP, *Klebsiella pneumoniae*; MDR, multi-drug resistant.

a UHF = university hospital, Frankfurt am Main.

b Highly virulent strains, associated with apoptosis^15^ and high cytotoxicity.^16^

### Identification of Key Metabolites From the Supernatants of Caco-2 Cells Infected With *Abaumannii* or *Kpneumoniae*

**Figure 2:**
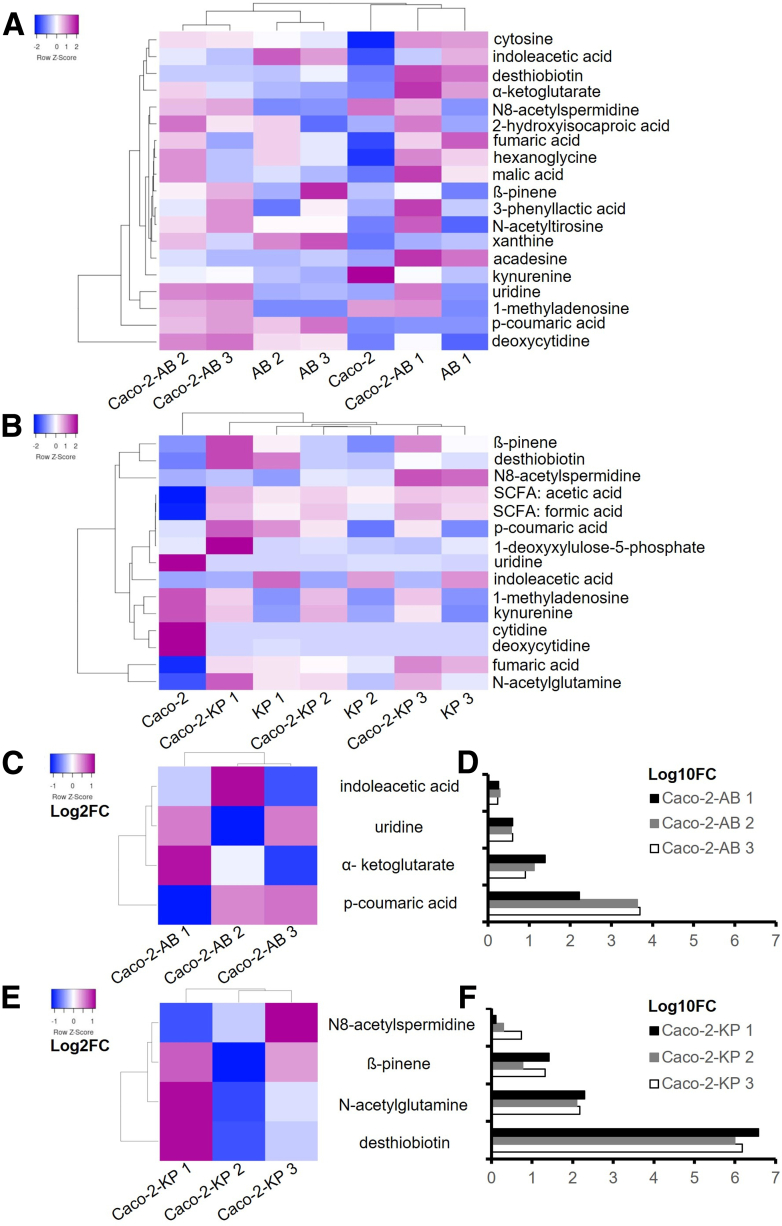
**Altered metabolites patterns in Caco-2 cells infected 6 hours with Abaumannii or Kpneumoniae compared with controls.** (*A* and *B*) Clustered heat map of mean relative metabolite abundances in supernatants of Caco-2 cells infected for 6 hours with *Abaumannii* (AB) or *Kpneumoniae* (KP) strains, respectively, to enhance interpretability. To identify metabolites increased due to bacterial infections, controls included supernatants from uninfected Caco-2 cells and bacterial cultures incubated without host cells for 6 hours under identical conditions. Metabolomics profiling was performed on 2 biological replicates per condition, from 2 independent experiments, each consisting of 3 replicates pooled at the supernatant level. Values were row-normalized using Z-score to emphasize relative differences across conditions. For further details, see Tables 2 and 3. (*C* and *E*) Clustered heatmaps showing log2-fold changes in selected metabolites levels, generated from mean relative abundances, following 6-hour infections of Caco-2 cells with *Abaumannii* (AB) or *Kpneumoniae* (KP), respectively. Fold changes were calculated by normalizing metabolite intensities to those in uninfected Caco-2 cell controls incubated under identical conditions, enabling identification of infection-associated metabolic increases. Z-score normalization was applied to each row of log2 fold changes matrices prior to clustering, to enhance pattern recognition across conditions. For further details, see Table 4. (*D* and *F*) Clustered heatmaps showing log10-fold changes in selected metabolites levels, generated from mean relative abundances, following 6-hour infections of Caco-2 cells with *Abaumannii* (AB) or *Kpneumoniae* (KP), respectively. Fold changes were calculated by normalizing metabolite intensities to those in uninfected Caco-2 cell controls incubated under identical conditions, enabling identification of infection associated metabolic increases. For further details, see Table 5.

To investigate the contribution of metabolites associated with gut bacterial infections to in vitro cytotoxicity, and their potential relevance to liver cell injury in ACLF, we performed metabolomics profiling of cell-free supernatants from Caco-2 cultures infected for 6 hours. For defining infection-driven metabolic changes, strict controls (supernatants from uninfected cells and bacteria cultured without host cells) were included (see Materials and Methods). All conditions were maintained under identical experimental parameters to ensure consistency and minimize confounding variables. Untargeted metabolomics analysis identified 1085 semi-polar metabolites. From these, 19 compounds from *A* *baumannii* infections and 15 from *K* *pneumoniae* infections annotated at levels 1 and 2a (indicating high structural confidence) were selected (Figure 1*D* and *E*; Tables 2 and 3). Targeted metabolomics was also performed to quantify short-chain fatty acids (SCFAs) in the supernatants (Figure 1*B* and *C*; Table 3). To compare metabolic shifts across conditions, clustered heatmaps of mean relative metabolite concentrations (to enhance interpretability) were generated (Figure 2*A* and *B*; Tables 2 and 3). Values were row-normalized using Z-score to emphasize relative differences across conditions. Eight metabolites were consistently elevated during infection, also known to be associated with mitochondrial dysfunction, oxidative stress, and reactive oxygen species (ROS) production.^18^, ^19^, ^20^, ^21^, ^22^, ^23^, ^24^, ^25^, ^26^, ^27^, ^28^ For *A* *baumannii*, these included uridine (pyrimidine metabolism^18^), α-ketoglutarate (Krebs cycle^19^), indoleacetic acid (tryptophan metabolism^20^), and p-coumaric acid (aromatic amino acid biosynthesis^21^). For *K* *pneumoniae*, selected metabolites were desthiobiotin (biotin metabolism^22^), β-pinene (terpenoid pathway^23^), N8-acetylspermidine (polyamine metabolism^24^), and N-acetylglutamine (nitrogen compound biosynthesis^25^). These compounds were visualized as log2 fold changes, generated from mean relative abundances to enhance interpretability, relative to uninfected Caco-2 controls, (Figure 2*C–E*; Table 4), and as log10 fold changes, generated from mean relative abundances to enhance interpretability, relative to uninfected Caco-2 cells controls (Figure 2*D–F*; Table 5). Z-score normalization was applied to each row of log2 fold changes matrices prior to clustering, to enhance pattern recognition across conditions. Although metabolite profiles differed between *K* *pneumoniae* and *A* *baumannii*, they remained consistent across strains within each species (Table 5), indicating that species identity, rather than strain variability, predominantly governs these metabolic signatures during infection.

**Table 2:** Mean Relative Abundance Values of *Abaumannii-*infected Caco-2 Cell Supernatants and Relative Controls

Relative abundances: *Abaumannii*-infected cells	Caco-2-AB 1	Caco-2-AB 2	Caco-2-AB 3	AB 1	AB 2	AB 3	Caco-2
Indole-3-acetic acid	76,607.00	82,786.50	73,767.50	109,041.50	128,144.50	113,697.50	42,808.50
2-hydroxyisocaproic acid	99,293.50	102,953.00	67,956.50	32,151.00	74,021.50	12,852.50	33,583.50
Cytosine	163,379.00	128,414.00	126,946.00	158,777.00	113,909.50	103,541.00	10,543.50
Kynurenine	23,687.50	14,363.50	21,772.00	−15,335.00	−15,168.50	−21,361.00	173,278.50
N8-acetylspermidine	60,349.54	58,447.54	64,967.54	−319.16	−976.62	−413.06	81,925.04
Xanthine	13,411.94	45,730.68	20,887.83	15,939.77	55,877.62	68,515.98	−1,050.61
Malic acid	54,096.50	43,971.00	24,628.00	35,189.50	27,676.00	22,813.50	14,484.00
Hexanoglycine	35,062.00	33,973.00	13,648.50	26,605.00	26,813.00	18,976.00	−4,628.50
Fumaric acid	16,213.37	17,248.87	6,929.87	24,026.37	16,163.37	11,706.87	−96.15
ß-pinene	22,975.74	24,865.24	30,445.74	8,853.24	13,727.74	44,205.74	16,043.24
N-acetyltirosine	3,595.50	2,232.50	2,990.50	16.00	1,899.86	1,856.00	319.49
3-phenyllactic acid	20,306.97	2,923.37	13,802.25	328.24	−7,211.90	5,965.14	−1,941.49
Acadesine	86,788.80	16,939.80	6,916.80	68,190.30	6,902.30	10,217.30	633.38
Desthiobiotin	220,537.00	51,984.00	54,720.00	192,553.50	47,925.00	81,939.50	−5,763.00
α-ketoglutaric acid	316,919.45	171,928.45	103,569.95	221,301.95	66,006.95	41,745.95	12,841.45
1-methyladenosine	257,051.68	220,336.18	245,406.68	1,487.50	790.95	759.44	243,730.18
Uridine	342,657.29	323,541.29	342,564.79	72,425.79	104,886.79	110,613.79	87,179.29
p-coumaric acid	11,587.50	297,981.00	343,301.50	4,872.50	282,136.00	411,424.50	69.50
Deoxycytidine	560,539.00	686,128.50	704,401.50	394,379.00	598,020.50	592,522.00	421,397.50

NOTE. Relative abundances of altered metabolite patterns in Caco-2 cells infected for 6 hours with *Abaumannii* compared with controls. Mean relative abundancies values of metabolite patterns from supernatants of Caco-2 cells infected for 6 hours with *Abaumannii* strains, compared with controls: supernatants from uninfected Caco-2 cells, supernatants from bacteria grown without host cells, incubated 6 hours; see Material and Methods.

AB, *Acinetobacter baumannii*.

**Table 3:** Mean Relative Abundance Values of *Kpneumoniae-*infected Caco-2 Cell Supernatants and Relative Controls

Relative abundances: *Kpneumoniae-*infected cells	Caco-2-KP 1	Caco-2-KP 2	Caco-2-KP 3	KP 1	KP 2	KP 3	Caco-2
Desthiobiotin	375,065.00	97,751.00	151,521.50	322,553.00	75,995.00	123,463.50	−6,323.50
ß-pinene	427,480.67	96,016.67	336,344.67	189,991.67	−1,061.91	163,032.67	16,134.17
N8-acetylspermidine	117,209.02	177,477.52	485,331.52	57,127.02	159,467.52	445,061.52	89,521.02
SCFA: formic acid	16.72	16.31	17.76	14.10	11.95	14.81	0.14
SCFA: acetic acid	2.55	2.32	2.40	2.13	2.06	2.30	0.01
1-deoxyxylulose-5-phosphate	4,465.46	1,504.75	1,128.40	1,360.12	1,237.46	1,569.55	1,574.43
p-coumaric acid	2,627.00	164.00	81.00	1,700.50	−3,233.00	−2,814.50	−1,023.50
Uridine	−462.80	392.58	1,069.43	368.96	1,492.03	−362.95	96,931.98
Kynurenine	109,968.50	124,280.00	87,730.00	−8,681.50	−6,952.00	−27,219.50	189,709.50
Indole-3-acetic acid	35,101.50	30,911.00	46,024.00	225,674.50	188,950.50	196,827.50	24,665.00
Deoxycytidine	1,312.00	1,143.00	−5,100.00	5,295.50	968.00	−2,393.00	486,259.00
Cytodine	1,637.97	1,787.97	−185.53	−2,115.67	−555.82	−1,779.03	379,416.97
1-methyladenosine	176,046.93	198,623.43	187,088.93	−710.43	−240.58	−1,349.88	303,978.93
N-acetylglutamine	855,019.00	552,261.00	652,604.00	532,288.00	304,535.00	397,813.00	4,348.50
Fumaric acid	498,206.91	452,212.91	644,051.41	485,621.91	378,458.91	579,523.91	−1,225.90

NOTE. Relative abundances of altered metabolite patterns in Caco-2 cells infected for 6 hours with *Kpneumoniae* compared with controls. Mean relative abundancy values of metabolite patterns from supernatants of Caco-2 cells infected for 6 hours with *Kpneumoniae* strains, compared with controls: supernatants from uninfected Caco-2 cells, supernatants from bacteria grown without host cells, incubated 6 hours; see Material and Methods.

KP, *Klebsiella pneumoniae*; SCFA, short-chain fatty acid.

**Table 4:** Log2-Fold Change of Metabolites of Interest in *Abaumannii* or *Kpneumoniae-*infected Caco-2 Cell Supernatants Compared With Uninfected Caco-2 Cells Control, Calculated From Mean Metabolite Abundances

Log2 fold change	Caco-2-AB 1	Caco-2-AB 2	Caco-2-AB 3
*Abaumannii*-infected cells			
Indole-3-acetic acid	0.84	0.95	0.79
Uridine	1.97	1.89	1.97
α-ketoglutarate	4.63	3.74	3.01
p-coumaric acid	7.38	12.07	12.27

	Caco-2-KP 1	Caco-2-KP 2	Caco-2-KP 3

*Kpneumoniae*-infected cells			
N-acetylglutamine	7.62	6.99	7.23
ß-pinene	4.73	2.57	4.38
N8-acetylspermidine	0.39	0.99	2.44
Desthiobiotin	21.84	19.90	20.53

NOTE. Log2-fold change values of selected metabolites derived from 6-hour Caco-2 infections with *Abaumannii* or *Kpneumoniae* strains, normalized to levels in uninfected Caco-2 cell controls incubated under identical conditions, calculated from mean metabolite abundances.

AB, *Acinetobacter baumannii*; KP, *Klebsiella pneumoniae*.

**Table 5:** Log10-Fold Change of Metabolites of Interest in *Abaumannii* or *Kpneumoniae*-infected Caco-2 Cell Supernatants Compared With Uninfected Caco-2 Cells Control, Calculated From Mean Metabolite Abundances

Log10FC metabolites	Caco-2-AB 1	Caco-2-AB 2	Caco-2-AB 3	Mean Caco-2 AB	SD Caco-2 AB
*Abaumannii*-infected cells					
Indole-3-acetic acid	0.25	0.29	0.24	0.26	0.026
Uridine	0.59	0.57	0.59	0.59	0.014
α-ketoglutarate	1.39	1.13	0.91	1.14	0.243
p-coumaric acid	2.22	3.63	3.69	3.18	0.832

	Caco-2-KP 1	Caco-2-KP 2	Caco-2-KP 3	Mean Caco-2 KP	SD Caco-2 KP
*Kpneumoniae-*infected cells					
N-acetylglutamine	2.29	2.10	2.18	2.19	0.096
ß-pinene	1.42	0.77	1.32	1.17	0.348
N8-acetylspermidine	0.12	0.30	0.73	0.38	0.317
Desthiobiotin	6.57	5.99	6.18	6.25	0.298

NOTE. Log10-fold change of selected metabolites of interest derived from 6-hour Caco-2 infections with *Abaumannii* or *Kpneumoniae* strains, normalized to levels in uninfected Caco-2 cell controls incubated under identical conditions, highlighting the association between metabolites and bacterial strains infections, calculated from mean metabolite abundances. Mean and standard deviations are depicted.

AB, *Acinetobacter baumannii*; KP, *Klebsiella pneumoniae*; SD, standard deviation.

Metabolite origin was inferred through comparative analysis of relative abundances across the 3 experimental conditions described above. In *A* *baumannii* infection experiments (Table 2), α-ketoglutarate and p-coumaric acid were highly secreted by bacteria grown without Caco-2 cells, with lower levels detected in uninfected Caco-2 cells and predominantly elevated levels in supernatants from infected Caco-2 cells, indicating a bacterial origin amplified during infection. Uridine was similarly secreted by both host cells and bacteria, but its levels were substantially higher in infected conditions, suggesting a synergistic contribution. Indoleacetic acid was primarily of bacterial origin, with lower levels in uninfected cells and elevated but slightly lower values in infected supernatants. In *K* *pneumoniae* infection experiments (Table 3), desthiobiotin, β-pinene, N8-acetylspermidine, and N-acetylglutamine were highly secreted by bacteria (except for ß-pinene in KP 2). These metabolites were lower in uninfected Caco-2 supernatants and showed predominant abundance during Caco-2 cell infections. These results indicate that, although certain metabolites, such as uridine, may reflect a host-derived response, the majority of metabolic signatures are of bacterial origin and amplified during host-pathogen interaction. This approach provides strong evidence for metabolite origin and supports the biological relevance of the infection-associated metabolic profiles observed.

### Supernatants From Caco-2 Cells Infected With *Abaumannii* or *Kpneumoniae* Exhibit Cytotoxic Effects on HepG2 Cells

**Figure 3:**
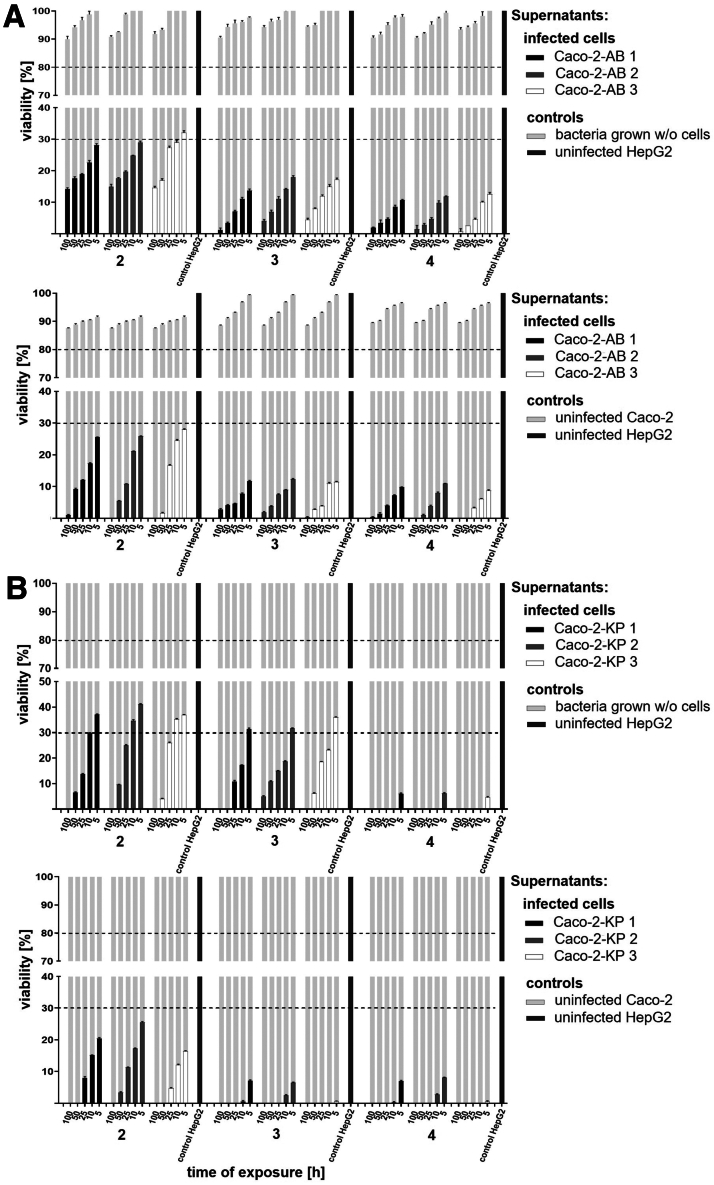
**Viability of HepG2 cells after stimulation with conditioned media derived from Caco-2 cells infected for 6 hours with (*A*) *Abaumannii* (AB) or (*B*) *Kpneumoniae* (KP) strains compared with controls.** Conditioned media from 6-hour infected-Caco-2 cells were added to HepG2 cultures at 5 different concentrations (100%, 50%, 25%, 10%, and 5%; diluted in fresh medium). Controls included conditioned media from uninfected Caco-2 cells, and from bacterial cultures incubated without host cells, were both prepared under identical 6-hour conditions, and added at matching concentrations. Untreated HepG2 cells served as negative controls. Viability of HepG2 cells was assessed via XTT assays after for 2, 3, and 4 hours of exposure. Bar charts depict results with supernatants taken from infections. *Black*, reference strains; *gray*, MDR strains; *white*, highly virulent strains. *Light gray bars* in the background represent control conditions. Each condition was tested in triplicates across 3 independent experiments. Values are means ± SD. All values differed from the appropriate controls with a statistical significance of *P* < .0001, determined using 2-way ANOVA analysis (not shown in graphics to maintain readability, given the extensive number of conditions considered).

In vivo, metabolites from the gut are transported to the liver via the portal vein.^3^, ^4^, ^5^ To simulate this physiological translocation, HepG2 liver cells were exposed to 5 concentrations of supernatants from 6-hour infected Caco-2 cells (see Materials and Methods). This exposure led to a dose-dependent reduction in HepG2 viability across all time points (Figure 3*A* and *B*) (*P* < .0001). Equivalent concentrations of control supernatants from uninfected Caco-2 cells and bacteria cultured without host cells (6-hour incubation) showed no cytotoxicity. These findings suggest that hepatotoxicity originates from infection-specific factors in the supernatants of infected Caco-2 cells that seem to contain a cytotoxic mixture of the previously identified metabolites.

### Metabolites Selected From Supernatants of Caco-2 Cells Infected With *Abaumannii* or *Kpneumoniae* Are Cytotoxic to HepG2-Cells

**Figure 4:**
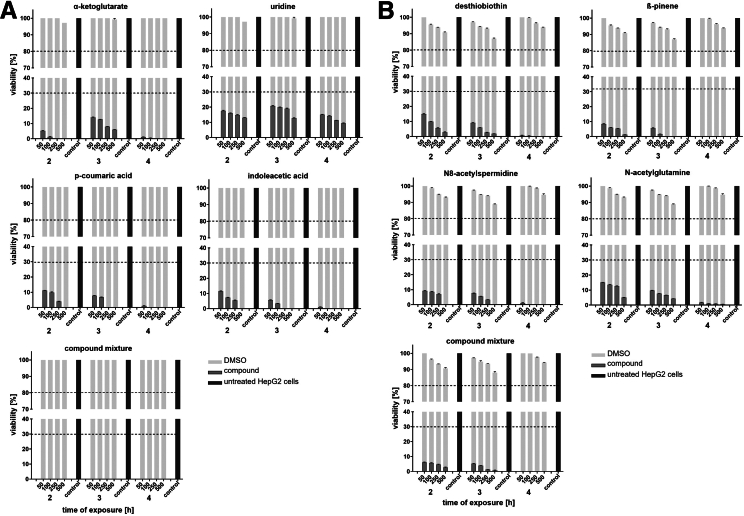
**Viability of HepG2 cells following stimulation with selected metabolites identified from Caco-2 cells infected 6 hours with (*A*) *Abaumannii* or (*B*) *Kpneumoniae* strains compared with controls.** HepG2 cells were exposed to individual metabolites and corresponding compound mixtures: α-ketoglutarate, uridine, p-coumaric acid, and indoleacetic acid (from *Abaumannii* infected-Caco-2 cells); desthiobiotin, β-pinene, N8-acetylspermidine, and N-acetylglutamine (from *Kpneumoniae* infected-Caco-2 cells). Each compound or mixture was applied at concentrations of 50, 100, 250, and 500 μg/mL diluted in fresh medium. Controls included HepG2 cells treated with DMSO (solvent control) at matching concentrations used for metabolite preparation, shown in *light gray bars* in the background. Untreated HepG2 cells served as negative control. Cell viability was evaluated using XTT assays after 2, 3, and 4 hours of exposure. Each condition was tested in triplicates across 3 independent experiments. Values are means ± SD. All values differed from the appropriate controls with a statistical significance of *P* < .0001, determined using 2-way ANOVA analysis (not shown in graphics to maintain readability, given the extensive number of conditions considered).

To elucidate the hepatotoxic effects of conditioned media, 8 metabolites identified in 6-hour infected-Caco-2 supernatants were selected for further analysis: α-ketoglutarate, uridine, p-coumaric acid, and indoleacetic acid from *A* *baumannii* infections; and desthiobiotin, β-pinene, N8-acetylspermidine, and N-acetylglutamine from *K* *pneumoniae*. HepG2 cells were exposed to 2 metabolite mixtures (each comprising the 4 respective metabolites associated with either bacterium), as well as to the individual compounds (all tested at 4 concentrations). Strict dimethyl sulfoxide (DMSO) solvent controls at matching concentrations were included to exclude matrix effects. A dose-dependent reduction in HepG2 viability was observed as early as 2 hours post-exposure for both individual compounds and mixtures (Figure 4*A* and *B*) (*P* < .0001), whereas DMSO controls had no effect. Notably, both mixtures induced stronger cytotoxicity than individual metabolites, suggesting additive detrimental effects, most pronounced with the *A* *baumannii*-derived compound mixture.

### Ex Vivo Validation of Infection-associated Metabolites in Sera From Patients With AD and ACLF

To investigate the potential association between hepatotoxic metabolites identified in vitro and ACLF, we performed ex vivo metabolomics analysis on serum samples from 2 independent patient cohorts. The Frankfurt cohort included healthy controls and sera from patients with ACLF of alcoholic origin (Table 6), whereas the Bonn/Münster cohort consisted of sera from patients with acutely decompensated liver cirrhosis, stratified by Model of End-stage Liver Disease (MELD) score (≤10 or >10) to assess disease severity (Table 7).

**Table 6:** Patient Characteristics of the Frankfurt Cohort (N = 20)

Characteristics	All patients (N = 20)	MELD ≤30 (n = 10)	MELD >30 (n = 10)	*P* value
Age, *y*	53.50 (45.75–58.00) (20)	55.00 (44.50–60.00) (10)	50.00 (46.25–55.75) (10)	1.000
Sex				1.000
Female	7 (35.0%) (20)	3 (30.0%) (10)	4 (40.0%) (10)	
Male	13 (65.0%) (20)	7 (70.0%) (10)	6 (60.0%) (10)	
AST, *IU/L*	115.00 (80.75–249.25) (20)	98.00 (70.25–365.00) (10)	140.00 (97.25–184.25) (10)	.393
ALT, *IU/L*	55.50 (34.50–142.50) (20)	49.50 (33.50–112.00) (10)	61.50 (41.25–171.50) (10)	.496
CRP, *mg/dL*	3.18 (2.00–4.76) (20)	3.29 (1.49–4.58) (10)	2.78 (2.11–4.81) (10)	.821
WBC, *× 1000/μL*	13.29 (9.06–17.31) (20)	14.88 (11.39–19.31) (10)	11.52 (8.82–13.97) (10)	.247
INR (median/range/sample size)	2.39 (1.84–2.54) (20)	1.88 (1.45–2.17) (10)	2.55 (2.52–2.95) (10)	.001
Child-Pugh class				1.000
Child B	1 (5.0%) (20)	1 (10.0%) (10)	0 (0.0%) (10)	
Child C	19 (95.0%) (20)	9 (90.0%) (10)	10 (100.0%) (10)	
Indication for TIPS				.474
NA	17 (85.0%) (20)	9 (90.0%) (10)	8 (80.0%) (10)	
Variceal bleeding	2 (10.0%) (20)	0 (0.0%) (10)	2 (20.0%) (10)	
Refractory ascites	1 (5.0%) (20)	1 (10.0%) (10)	0 (0.0%) (10)	
CLIF-C ACLF score	54.43 (46.36–59.07) (20)	51.29 (45.16–56.61) (10)	54.57 (50.46–62.65) (10)	.436
ACLF grade				.195
Grade 1	6 (30.0%) (20)	5 (50.0%) (10)	1 (10.0%) (10)	
Grade 2	9 (45.0%) (20)	3 (30.0%) (10)	6 (60.0%) (10)	
Grade 3	5 (25.0%) (20)	2 (20.0%) (10)	3 (30.0%) (10)	
Etiology of cirrhosis				NA
Alcohol	20 (100.0%) (20)	10 (100.0%) (10)	10 (100.0%) (10)	
Bacterial infections				1.000
No	5 (25.0%) (20)	3 (30.0%) (10)	2 (20.0%) (10)	
Yes	15 (75.0%) (20)	7 (70.0%) (10)	8 (80.0%) (10)	

NOTE. Data are presented as number (%) (sample size) or median (range) (sample size).

ACLF, acute-on-chronic liver failure; ALT, alanine aminotransaminase; AST, aspartate aminotransaminase; CRP, C-reactive protein; INR, International Normalized Ratio; MELD, Model of End-stage Liver Disease; NA, not available; TIPS, transjugular intrahepatic portosystemic shunt; WBC, white blood cell count.

**Table 7:** Patient Characteristics of the Bonn/Münster Cohort (N = 32)

Characteristics	MELD ≤10	MELD >10	*P* value
	n = 13	n = 19	
Age, *y*	56 (34–81)	59 (51–72)	.166
Male sex	5 (38.5)	13 (68.4)	.188
AST, *IU/L*	30.5 (±71.4)	46.0 (±26.0)	.128
ALT, *IU/L*	27.0 (±61.0)	20.0 (±15.6)	.290
CRP, *mg/dL*	5.5 (±29.0)	15.3 (±15.4)	.301
WBC, *× 1000/μL*	8.7 (±7.6)	6.7 (±3.0)	.514
INR	1.1 (±0.2)	1.2 (±0.3)	.184
Child-Pugh class			
Child A	8 (61.5)	0.0	.0001
Child B	4 (30.8)	16 (84.2)	.0035
Child C	0.0	2 (10.5)	.502
Indication for TIPS (repeated or high-risk variceal bleeding/refractory or recurrent ascites/others^a^)	4/6/3	2/16/1	.072
CLIF C scores, median (±SD; no.)			
CLIF C AD	44.5 (±6.6; 12)	49.0 (±5.6; 13)	.209
CLIF C ACLF	35.0 (±0.0; 1)	42.5 (±3.1; 6)	.207

NOTE. Data are presented as number (%), median (range), or mean ± standard deviataion.

ACLF, acute-on-chronic liver failure; ALT, alanine aminotransaminase; AST, aspartate aminotransaminase; CRP, C-reactive protein; INR, International Normalized Ratio; MELD, Model of End-stage Liver Disease; NA, not available; TIPS, transjugular intrahepatic portosystemic shunt; WBC, white blood cell count.

a Portal vein thrombosis, TIPS dysfunction.

#### Frankfurt cohort

**Figure 5:**
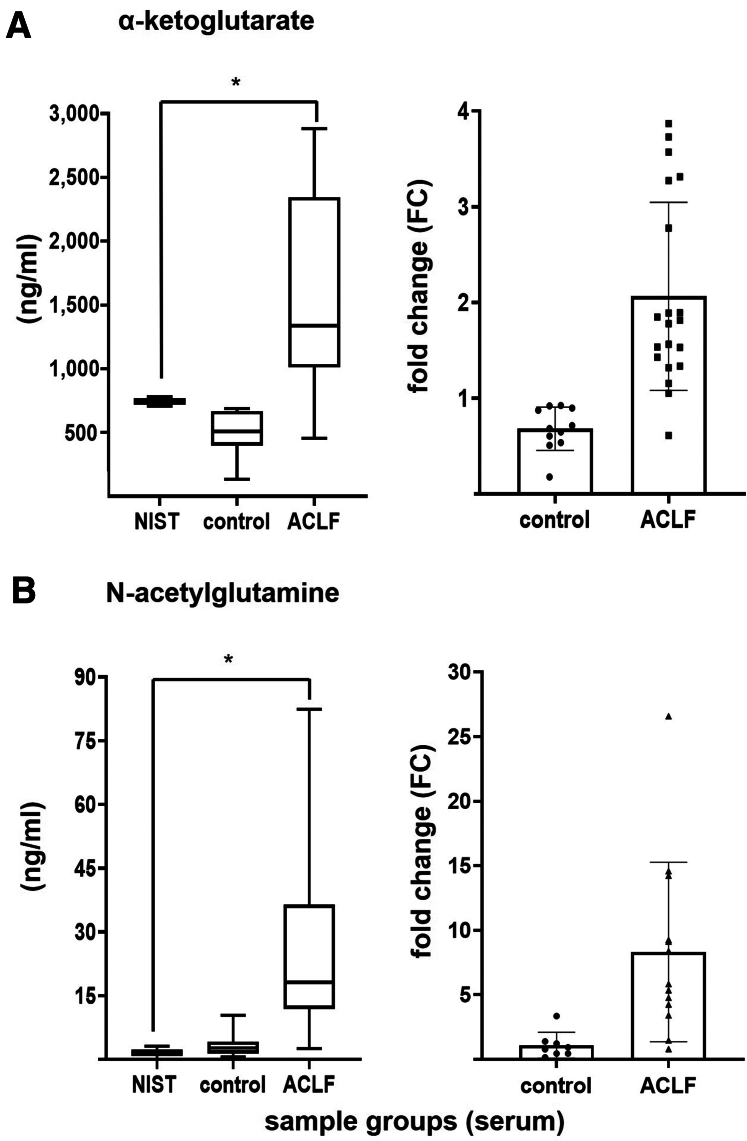
**Metabolomics analysis of ACLF patient sera: Frankfurt cohort.** (*A*) *Left*: Concentration of α-ketoglutarate (ng/mL) across sample group’s sera (reference standard NIST, healthy controls, patients with ACLF). Median is represented in the center, minimum and maximum values at whiskers extremities. Statistical 1-way ANOVA analysis revealed significant difference among the means (∗*P* = .0002). *Right*: Fold change of α-ketoglutarate. Means and SDs are depicted. Control data points: n = 11; ACLF data points: n = 20. (*B*) *Left*: Concentration of N-acetylglutamine (ng/mL) across sample group’s sera (reference standard NIST, healthy controls, patients with ACLF). Median is represented in the center, minimum and maximum values at whiskers extremities. Statistical 1-way ANOVA analysis revealed significant difference among the means (∗*P* = .0153). *Right*: Fold change of N-acetylglutamine. Control data points: n = 8; ACLF data points: n = 13.

In the Frankfurt cohort, semi-quantitative analysis was performed on sera from patients with ACLF and healthy controls using high-resolution mass spectrometry. To ensure better inter-study comparability, all results were normalized to the Standard Reference Material (SRM) 1950 National Institute of Standards and Technology (NIST) reference material, adding consistency and reliability to the data. Among the 8 hepatotoxic metabolites identified through in vitro experiments (see above), α-ketoglutarate and N-acetylglutamine were also significantly elevated in patient serum samples, underscoring their translational and biological relevance. In particular, α-ketoglutarate, vital for Krebs cycle regulation,^19^ was consistently increased in sera from patients with ACLF (n = 20) compared with controls (n = 11) and NIST (Figure 5*A*) (*P* = .0002). Similarly, N-acetylglutamine, fundamental for nitrogen clearance,^25^ was notably increased in sera from patients with ACLF (n = 13) compared with healthy controls (n = 8) and NIST (Figure 5*B*) (*P* = .0153).

#### Bonn/Münster cohort

**Figure 6:**
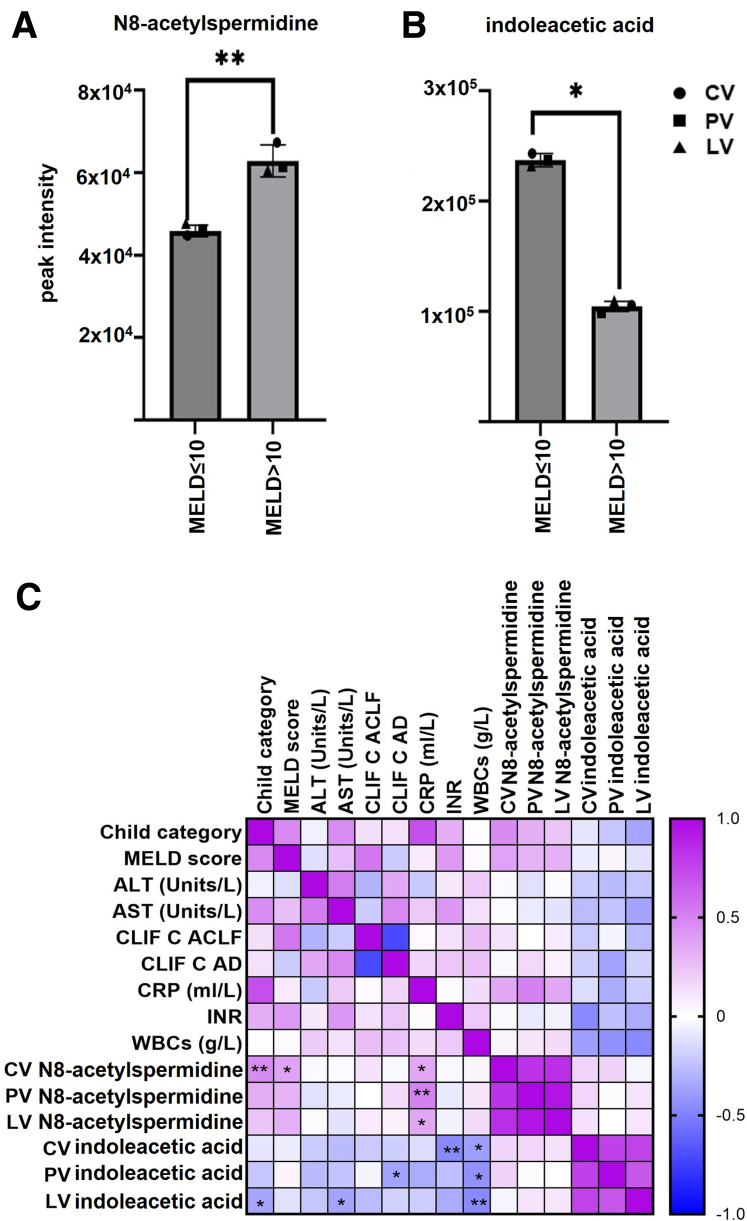
**Metabolomics analysis of ACLF patient sera: Bonn/Münster cohort.** (*A*) Serum peak intensities in TIPS cohort patients of N8-acetylspermidine, grouped by different MELD score levels (for MELD >10, n = 19; for MELD ≤10, n = 13), to determine differences across severity of the disease. Statistical significance was determined using 2-way ANOVA. ∗*P* < .05; ∗∗*P* < .01. (*B*) Serum peak intensities of indoleacetic acid in TIPS cohort patients, grouped by different MELD score levels, to determine differences across severity of the disease. Statistical significance was determined using 2-way ANOVA testing. (*C*) Spearman correlations of N8-acetylspermidine and indoleacetic acid with different disease markers in TIPS cohort patients. ∗*P* < .05; ∗∗*P* < .01.

Untargeted metabolomics of serum samples from acutely decompensated cirrhotic patients revealed elevated peak intensities of N8-acetylspermidine and indoleacetic acid across portal, hepatic, and cubital veins. Patients were stratified by MELD score (≤10 vs >10) to assess disease severity. N8-acetylspermidine levels were significantly higher in patients with MELD >10 (n = 19) compared with patients with MELD ≤10 (n = 13; *P* < .01) (Figure 6*A*), whereas indoleacetic acid levels were consistently lower in the MELD >10 group across all compartments (Figure 6*B*) (*P* < .05). These findings suggest that N8-acetylspermidine accumulates with worsening liver function, potentially contributing to toxicity even at low concentrations, reflecting polyamine pathway dysregulation.^24^^,^^26^ In contrast, indoleacetic acid inverse trend may indicate disrupted gut–liver microbial co-metabolism in advanced cirrhosis.^20^^,^^27^ Spearman correlations (biochemistry and disease scores) showed N8-acetylspermidine positively correlated with Child-Pugh category, MELD score, and the inflammatory marker C-reactive protein (CRP), linking it to disease severity and systemic inflammation. Indoleacetic acid negatively correlated with white blood cell count (WBCs), clotting ability (International Normalized Ratio [INR]), acute decompensation score (CLIF C AD), liver injury (aspartate aminotransaminase [AST]) and decompensation severity (Child-Pugh category), reinforcing its microbial origin and emphasizing the role of gut-liver interactions in acutely decompensated liver cirrhosis (Figure 6*C*).

## Discussion

The present study identifies distinct metabolites associated with gut bacterial infections and hepatotoxicity which may contribute to ACLF pathophysiology. Using an in vitro model of gut epithelial cell infection with *A* *baumannii* or *K* *pneumoniae* and subsequent metabolomics analysis, we uncovered species-specific metabolic signatures. Eight metabolites were selected for further analysis, all of which demonstrated toxic effects on HepG2 cells. Four, α-ketoglutarate, N-acetylglutamine, indoleacetic acid, and N8-acetylspermidine, were significantly elevated in sera from patients with AD and ACLF from 2 independent cohorts (Frankfurt and Bonn/Münster), potentially linking infection-induced metabolic shifts to ACLF.

To uncover infection-associated metabolic alterations^9^^,^^10^^,^^17^ and to mimic gut–liver crosstalk relevant to ACLF,^3^, ^4^, ^5^, ^6^ we performed metabolomic profiling on supernatants collected from Caco-2 cells infected with *A* *baumannii* or *K* *pneumoniae* (Figure 2*A* and *B*), based on our rationale for strain selection (see Introduction). These analyses revealed specific metabolic changes associated to infections, from which we prioritized compounds previously linked to oxidative stress and ROS production (Table 8).^18^, ^19^, ^20^, ^21^, ^22^, ^23^, ^24^, ^25^, ^26^, ^27^, ^28^ In *A* *baumannii* infections, α-ketoglutarate, uridine, p-coumaric acid, and indoleacetic acid were prominent; in *K* *pneumoniae*, desthiobiotin, ß-pinene, N8-acetylspermidine, and N-acetylglutamine were identified (Figure 2*C–F*). To assess their hepatotoxic potential, HepG2 cells were subsequently exposed to the infected intestinal supernatants harboring these compounds (Figure 3), individual metabolites, and compound mixtures (Figure 4). Both approaches induced dose- and time-dependent reductions in HepG2 viability, suggesting synergistic toxicity and reflecting mechanisms relevant to liver cell damage. These results corroborate earlier studies, showing that high levels of these specific metabolites can display cytotoxicity. In fact, p-coumaric acid induces ROS-mediated mitochondrial dysfunction, leading to apoptosis in neural cells,^29^ whereas indoleacetic acid exhibits broad toxicity across cell lines and animal models.^30^ Excess of α-ketoglutarate promotes oxidative stress and destabilizes hypoxia-inducible factor 1-alpha (HIF-1α), impairing differentiation and inducing cell death in mesenchymal stromal precursors; in vivo, high doses cause diarrhea, weight loss, and anemia in Wistar rats.^28^^,^^31^ N8-acetylspermidine also exhibits dose-dependent cytotoxicity when administered intraperitoneally in mice.^32^

**Table 8:** Hepatotoxic Metabolites Identified In Vitro and Validated Ex Vivo

Metabolites identified in vitro	Description	Metabolites validated ex vivo
*Abaumannii* infection experiments		
α-ketoglutarate	Critical Krebs cycle intermediate, connected to mitochondrial dysfunction and oxidative stress,^10^^,^^19^ potential nutrient source for bacteria,^19^ toxic effects at higher concentrations^28^^,^^31^	Yes^a^
Indoleacetic acid	Product of tryptophan metabolism, implicates gut microbiota dysbiosis and gut-liver axis imbalance,^10^^,^^20^^,^^27^^,^^33^ toxic effects at higher concentrations^30^	Yes^b^
p-coumaric acid	Related to biosynthesis of aromatic amino acids, antioxidant and antitumor properties through modulation of cell cycle-related proteins and apoptosis-related proteins,^21^ toxic effects at higher concentrations^29^	No
Uridine	Related to pyrimidine metabolism, involved in energy metabolism and redox balance,^18^ potential nutrient source for bacteria^34^	No
*Kpneumoniae* infection experiments		
Desthiobiotin	Biotin precursor, vital role in synthesis of biotin coenzyme, crucial for various metabolic processes, mainly stored in liver^22^	No
N8-acetylspermidine	Derivate of polyamine pathway, disrupts cellular homeostasis by inducing oxidative stress, DNA damage, and activating apoptotic and autophagy pathways,^24^^,^^26^ toxic effects at higher concentrations and pathogenicity contribution^32^^,^^35^	Yes^b^
N-acetylglutamine	Allosteric activator of carbamoylphosphate synthetase I in urea cycle, reflects impairments in ammonia detoxification and nitrogen clearance, aligning with hyperammonemia^25^^,^^36^	Yes^a^
ß-pinene	Involved in terpenoids metabolic pathway^23^	No

a Validated in the Frankfurt cohort.

b Validated in the Bonn/Münster cohort.

Beyond direct cytotoxicity, these compounds may additionally influence host–pathogen dynamics: indoleacetic acid, synthesized by various bacteria including *Acinetobacter spp,* enhances stress tolerance and biofilm formation, key to bacterial survival and pathogenicity.^33^ N8-acetylspermidine modulates polyamine metabolism, aiding bacterial adaptation and immune evasion.^35^ α-Ketoglutarate and uridine may serve as nutrient sources, supporting pathogen survival.^18^^,^^28^ Collectively, the consistency of our findings across experimental setups reinforces the robustness of our conclusions, linking *A* *baumannii* and *K* *pneumoniae* infection-associated metabolites to alterations in liver cell function.^4^^,^^7^^,^^15^^,^^18^

**Figure 7:**
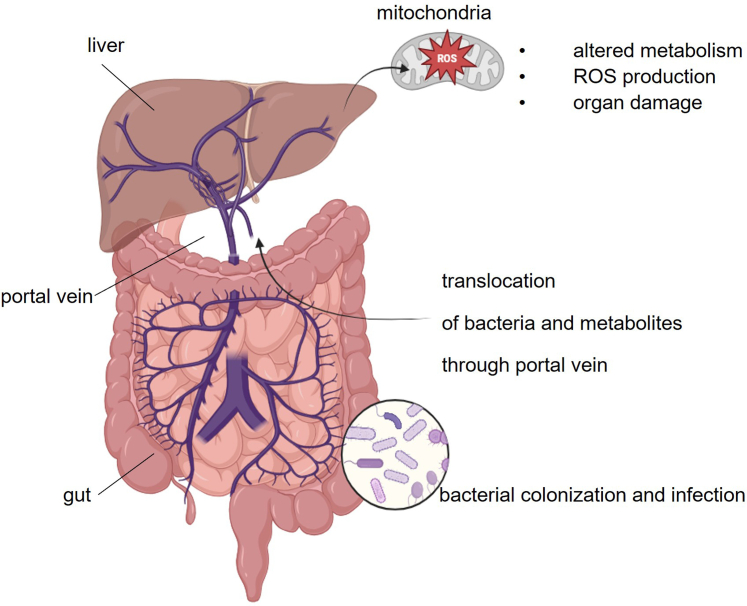
**Schematic representation of gut-liver axis imbalance as potential trigger of ACLF.** Gut barrier impairment may facilitate the translocation of bacteria and microbial metabolites into the portal vein, thereby exacerbating hepatic injury. Within the liver, these translocated products, particularly those associated with bacterial infections, may contribute to mitochondrial oxidative stress, leading to excessive ROS production and metabolic dysregulation. This cascade of events could accelerate hepatocyte damage and contribute to the progression of ACLF (created in BioRender.com [https://BioRender.com/pk055v4]).

The selection of this model, which involves infecting Caco-2 cells (intestinal epithelial cells) followed by the analysis of the effects induced by infection-associated metabolites on HepG2 cells (hepatocytes), is based on the observation that bacterial pathogens, particularly MDR strains under antibiotic pressure, initially colonize the intestine. The translocation of infection-associated hepatotoxic metabolites to the liver may represent the critical pathogenic mechanism underlying bacterially triggered ACLF in vivo. Notably, prior studies have demonstrated that bacterial infections can modulate the expression and localization of tight junction proteins in Caco-2 cells, leading to enhanced paracellular permeability and facilitating the translocation of bacteria or their metabolites across the epithelial barrier.^37^, ^38^, ^39^ It is therefore plausible to speculate that these bacteria, or their metabolites, might translocate from the intestinal microbiota to the liver via the portal vein, especially under conditions of gut barrier disruption. Furthermore, the in vitro experimental setup used, based on analyzing the bacterial metabolic signatures during their interaction with intestinal cells, lies at the intersection of intestinal cellular infection, gut lumen colonization, and dysbiosis, characterized by the overgrowth of specific bacterial species. In patients (eg, patients with ACLF), this results in direct contact between epithelial cells and the respective bacteria, potentially compromising gut barrier integrity (as discussed above) and thereby promoting the translocation of bacterial metabolites into the portal circulation through a “leaky” gut.^40^^,^^41^ Our in vitro metabolomics analysis demonstrated that the selected metabolites are predominantly derived from the bacteria, with this effect even more pronounced under co-culture conditions (see Tables 2 and 3), suggesting that these compounds could be produced in vivo during intestinal overgrowth. This mechanism may be particularly relevant in ACLF, where increased intestinal permeability promotes the systemic dissemination of microbial products, contributing to the deterioration of liver function.^40^^,^^41^ This process, potentially exacerbated by mitochondrial dysfunction and excessive ROS production in liver cells, may reflect metabolic imbalances possibly connected to ACLF^1^^,^^2^^,^^15^^,^^16^ (Figure 7).

To translate in vitro experimental findings into clinical relevance across distinct patient populations and severity profiles, we applied metabolomics analysis on serum samples from 2 independent cohorts. In the Frankfurt cohort, derived from a prospective study, α-ketoglutarate (Figure 5*A*; in Table 8) and N-acetylglutamine (Figure 5*B*; in Table 8), were highly increased in sera from patients with ACLF of alcoholic origin compared with healthy controls. These metabolites are linked to TCA cycle dysregulation^10^ and hyperammonemia-related nitrogen clearance disruptions,^36^ respectively, both central mechanisms in ACLF. In the Bonn/Münster cohort, N8-acetylspermidine (Figure 6*A*; Table 8) and indoleacetic acid (Figure 6*B*; Table 8) were detected in sera from AD cirrhotic patients, stratified by MELD score to assess disease severity, exhibiting distinct trends. N8-acetylspermidine accumulates as liver function deteriorates, potentially exerting toxic effects even at lower concentrations.^24^^,^^26^ In contrast, the indoleacetic acid shift may indicate disruptions in gut–liver microbial co-metabolism in advanced cirrhosis,^20^^,^^27^ altering metabolite production as decompensated cirrhosis advances. Both compounds have been previously associated with sepsis-related ACLF, reinforcing their relevance to infection-driven metabolic dysregulation.^10^ It is important noting that the patients in our study had documented infections but did not meet criteria for sepsis, which would have been a contraindication for transjugular intrahepatic portosystemic shunt (TIPS), to prevent further clinical deterioration. Our data suggest that our metabolites panel, although released in vitro following infection with specific bacterial species (*A* *baumannii* or *K* *pneumoniae*), is generally detectable in vivo in patients with various bacterial infections, supporting a model of how infections impair liver function (see Figure 7).

The partial overlap between the 8 candidate metabolites identified in vitro and those detected in patient sera (4 of which were significant) and the variation across cohorts reflects the complexity of human physiology. Although our in vitro model is well-suited to study localized bacteria-epithelial interactions along the gut-liver axis,^39^ it does not capture the full range of systemic influences, such as hepatic dysregulation and immune modulation. In vivo, metabolites from bacterial colonization or infection may be rapidly altered, diluted, or masked by competing pathways, or amplified through inflammatory loops, depending on the host’s condition. This layered complexity might explain why some metabolites are detectable in vitro but not in patient blood, highlighting the need to integrate cellular models with clinical data to better understand infection-associated metabolic shifts.

The divergence in metabolite profiles between the Frankfurt and Bonn/Münster cohorts further illustrates this point. In Frankfurt, elevated levels of α-ketoglutarate and N-acetylglutamine suggest mitochondrial dysfunction and impaired nitrogen metabolism, consistent with hepatic energy failure and urea cycle disruption.^10^^,^^36^ In contrast, the Bonn/Münster cohort exhibited increased levels of N8-acetylspermidine and indoleacetic acid, linked to immune activation and microbiome-derived signaling, respectively.^20^^,^^24^^,^^26^^,^^27^ These distinct profiles reflect different biological axes (hepatic metabolic collapse vs gut-liver immune crosstalk), yet both contribute to systemic deterioration in ACLF. Importantly, these pathways may interact: inflammation can worsen mitochondrial stress, and nitrogen imbalance can amplify immune dysfunction. This dual-pathway perspective underscores the multifactorial nature of ACLF and supports the use of metabolomics to capture converging disease mechanisms.

Our strategy bridges the mechanistic precision of in vitro infection models with the biological complexity of ex vivo patient data. Despite biological variability and systemic complexity of ACLF, 4 of 8 hepatotoxic metabolites were validated across both platforms, supporting their candidacy as potential biomarkers and therapeutic targets.^1^^,^^2^^,^^10^, ^11^, ^12^ These metabolites, derived from *K* *pneumoniae* or *A* *baumannii* infections, reflect conserved species-specific profiles that persist across reference, MDR, and highly virulent strains. This consistency suggests that species identity, rather than strain-level variation, shapes the metabolic landscape during infection. The clinical relevance of these metabolites originating from bacterial infections underscores the potential for targeted interventions in ACLF and personalized treatment strategies.

## Materials and Methods

Table 9 compiles specifications of essential reagents and methodological parameters applied throughout the study.

**Table 9:** CTAT With Material Specifications

Antibodies		
Name	Supplier	Cat. No.
Rabbit anti-*Abaumannii* IgG	Antigen produced by us; sera produced by Kaneka Eurogentec S.A.	
Rabbit anti-*Kpneumoniae* IgG	Antigen produced by us; sera produced by Kaneka Eurogentec S.A.	
Alexa 488 conjugated anti-rabbit IgG	Dianova	154288
Alexa 647 conjugated anti-rabbit IgG	Dianova	151912

Cell lines				
Name	Supplier	Passage (p)	PCR mycoplasma	Authentication
HepG2	DSMZ, German Collection of Microorganisms and Cell Culture	p15-p16	Mycoplasma free	STR authentication
Caco-2	DSMZ, German Collection of Microorganisms and Cell Culture	p43-p44	Mycoplasma free	STR authentication

Bacterial strains		
Name	Supplier	Characteristics
AB 1: *Abaumannii* 19606	ATCC, American Type Culture Collection	Reference strain
AB 2: *Abaumannii* 1372	University Hospital Frankfurt am Main	Multidrug-resistant bacteria, patient isolate
AB 3: *Abaumannii* 2778	University Hospital Frankfurt am Main	Multidrug-resistant bacteria and highly virulent, patient isolate
KP 1: *Kpneumoniae* 700603	ATCC, American Type Culture Collection	Reference strain
KP 2: *Kpneumoniae* 5989	University Hospital Frankfurt am Main	Multidrug-resistant bacteria, patient isolate
AB 3: *Kpneumoniae* 1664	University Hospital Frankfurt am Main	Multidrug-resistant bacteria and highly virulent, patient isolate

Biological samples			
Description	Source	Storage conditions	Ethics and approval
Frankfurt cohort: sera from patients with ACLF and healthy patient sera	University Hospital Frankfurt am Main	−80°	Ref. 20-653 and 329-10, signed consent of all participants following the Declaration of Helsinki
Münster cohort: AD and sera from patients with ACLF	University Clinic Bonn	−80°	029/13 and 203/13, signed consent of all participants following the Declaration of Helsinki

Metabolites		
Name	Supplier	Source
α-ketoglutarate	Sigma-Aldrich	BCCH3270
Indoleacetic acid	Sigma-Aldrich	SLCL0192
p-coumaric acid	Sigma-Aldrich	BCCJ1325
Uridine	Sigma-Aldrich	BCCK4390
Desthiobiotin	Sigma-Aldrich	SLCP2674
N-acetylglutamine	Sigma-Aldrich	129K2577V
ß-pinene	Roth	7106.1
N8-acetylspermidine	Chemos	A0063942

Software		
Name	Manufacturer	Version
Prism	GraphPad	8
Microsoft Office	Microsoft	2021
XCalibur	Thermo Fisher Scientific	4.4
TraceFinder	Thermo Fisher Scientific	5.1
Workflow4Metabolomics	MetaboHUB	3.0
Skyline	MacCoss, University of Washington	22.2.0.255
MatLab	MathWorks	2022b

AB, *Abaumannii*; ACLF, acute-on-chronic liver failure; IgG, immunoglobulin G; KP, *Kpneumoniae*; PCR, polymerase chain reaction.

### Bacterial Strains and Cell Lines

Infection experiments were performed using 6 strains of *A* *baumannii* and *K* *pneumoniae* (Table 1), including reference strains AB 1(*A* *baumannii* ATCC 19606^T^) and KP 1 (*K* *pneumoniae* ATCC 700603), purchased from DSMZ (German Collection of Microorganisms and Cell Culture). Bacteria were cultured on Columbia blood agar (CBA) plates (Thermo Fisher Scientific). The human immortalized HepG2 (hepatocarcinoma) and Caco-2 (adenocarcinoma) cell lines were acquired from DSMZ and cultivated in 75-cm^2^ cell culture flasks using Dulbecco’s Modified Eagle Medium (DMEM;41965-039, Gibco) supplemented with 10% fetal calf serum (FCS; Sigma-Aldrich) without antibiotics. Culture flasks were incubated at 37°C in 5% CO_2_ and 95% relative humidity. Passage numbers (p) were standardized across experiments: p15–p16 for HepG2 and p43–p44 for Caco-2 cells.

### Infection of Caco-2 Cells With *Abaumannii* or *Kpneumoniae*

For infection experiments, Caco-2 cells were seeded into collagene-coated 24- or 96-well plates (Collagen G 0,4%, Matrix BioScience GmbH, diluted 1:40 in Dulbecco's phosphate-buffered saline no magnesium and no calcium (DPBS); Gibco) at a density of 5 × 10^5^ or 80 × 10^4^ cells/mL. Bacterial strains (Table 1) were cultured in lysogeny broth (LB; Becton Dickinson) at 37°C, starting from optical density at 600 nm (OD_600_) of 0.05 and grown to OD_600_ of 0.2. Cultures were centrifuged (5000 × g for 15 minutes), washed with DPBS and resuspended in DMEM without antibiotics or FCS. Final multiplicity of infection (MOIs) was 100 for *A* *baumannii* and 1 for *K* *pneumoniae*. Cell culture plates were centrifuged for 3 minutes at 300 × g (to ensure bacterial contact to the surface of the cell layer) and incubated for 6 hours at 37°C, 5% CO_2_ and relative humidity of 95%. Viable bacteria were quantified by serial dilutions on CBA plates (Thermo Fisher Scientific), with colony-forming units (CFU) counted after 24 hours at 37°C.

### Generation of Anti-*Kpneumoniae* and *Abaumannii* Antibodies

Rabbit anti-*A* *baumannii* immunoglobulin Gs (IgGs) were generated using a mix of 6 clinical isolates (derived from international clusters 1, 2, 4, 6, 7, 8^15^; including AB 2 from Table 1) and 2 reference strains (ATCC 17978, and AB 1: ATCC 19606^T^ from Table 1). Rabbit anti-*K* *pneumoniae* IgGs were produced using 6 clinical isolates (including KP 2 and KP 3 from Table 1) and one reference strain (KP 1). Bacteria were cultured in LB broth (OD_600_ = 0.7), washed 3 times with DPBS and inactivated with 10% formalin (VWR International GmbH) for 2 hours at 37°C. Two rabbits were immunized on days 0, 7, 10, and 18 with 5.75 × 10^7^ inactivated bacteria per dose (Kaneka Eurogentec SA), and serum was collected on day 28. The reactivity of immune and pre-immune sera was evaluated by immunofluorescence at a dilution of 1:400 in DPBS.

### Double Immunofluorescence Staining of Infected Caco-2 Cells

Caco-2 cells were seeded on 12-mm coverslips (Karl/Hecht GmbH) in 24-well plates and afterwards infected for 6 hours with *A* *baumannii* (MOI 100) or *K* *pneumoniae* (MOI 1) in DMEM. Coverslips were washed 3 times with DPBS between staining steps. Cells were fixed using 3.75% paraformaldehyde (Sigma Aldrich; diluted in DBPS, pH 7.4) and blocked with 0.2% bovine serum albumin (BSA; Sigma-Aldrich) in DPBS for 15 minutes at room temperature (RT).

Primary antibodies (1:200, rabbit-anti-*A* *baumannii*; 1:400, rabbit anti-*K* *pneumoniae*) and secondary antibodies (anti-rabbit Alexa Fluor 488 IgG, 1:200, Dianova), were applied for 1 hour at RT. Cells were then permeabilized with 0.2% Triton X-100 (AppliChem GmbH) for 15 minutes at RT and re-blocked. Intracellular bacteria were stained with the same primary antibodies and Alexa Fluor 647-conjiugated secondary antibody (anti-rabbit IgG, 1:200, Dianova). Tetramethylrhodamine (TRITC)-phalloidin (Sigma Aldrich) was used for actin cytoskeleton staining (1 hour, RT). DNA was stained with 4′,6-diamidino-2-phenylindole (DAPI; Merck) for 10 minutes. Coverslips were finally mounted on glass slides using Fluorescence Mounting Medium (Agilent Technologies). Fluorescence microscopy analysis was performed with the microscope Zeiss Axio Imager 2 (Zeiss) equipped with a Spot RT3 camera (Diagnostic Instruments Inc) and operated by VisiView V.2.0.5 (Visitron Systems). Images were aquired with 63× objective.

### Untargeted Metabolomics for Semi-polar Metabolites Identification

Two biological replicates per condition were generated from two independent experiments, each consisting of 3 replicates pooled at the supernatant level. Samples were collected from Caco-2 cells infected for 6 hours with *A* *baumannii* or *K* *pneumoniae*, alongside controls under identical conditions (uninfected Caco-2 cells and bacteria cultured without host cells) to ensure infection-specific effects. Samples were centrifuged at 4°C (20.817 × g for 10 minutes), filter-sterilized (0.22 μm pore size; Thermo Fisher Scientific), collected in low-binding tubes (Sorenson, BioScience), and stored at −80°C. Metabolomics were performed by cmbio. Samples (10 μL) were diluted in 100-μL eluent A (water, 10 mM ammonium formate, 0.1% formic acid) containing stable isotope-labeled metabolites. Analysis was conducted with ultra-high-performance liquid chromatography (UHPLC) Vanquish coupled to a Q Exactive HF Hybrid Quadrupole-Orbitrap (Thermo Fisher Scientific). Electrospray ionization was performed in positive and negative ionization mode under polarity switching. Data were processed using Compound Discoverer 3.3 (Thermo Fisher Scientific) and Skyline 22.2 (MacCoss Lab Software) for peak picking and feature grouping, followed by curation in MATLAB (2022b). Compound annotations followed 4 confidence levels (level 1: retention times [±0.2 minutes], accurate mass [± 3 ppm], and tandem mass spectrometry [MS/MS] spectra [match factor >60] vs in-house standards; level 2a: retention times [±0.2 minutes], and accurate mass [±3 ppm] vs in-house standards; level 2b: accurate mass [±3 ppm], and MS/MS spectra vs in-house standards and mzCloud [Thermo Fisher Scientific] at a match factor >60; level 3: identification by accurate mass alone [±3 ppm], based on the Human metabolome database [v 5.0]). Co-eluting isotopes with indistinguishable retention and fragmentation were manually integrated and reported as compound groups (eg, hexoses, sugar alcohols). This untargeted profiling approach enabled broad, transparent coverage of relative metabolite concentrations in supernatants.

### Targeted Metabolomics for SCFA Identification

SCFAs were quantified from 30 μL of cell culture supernatants samples (conditions described above), acidified using an aqueous solution of hydrochloric acid and deuterium-labelled internal standards (36 μL, 0.2 M HCl). Analysis was carried out on a high polarity column (Zebron ZB-FFAP, GC Cap. Column 30 m × 0.25 mm × 0.25 μm) installed in a gas chromatography (GC, 7890B, Agilent) coupled with a time-of-flight mass spectrometer (Pegasus BT, LECO). The system was controlled by ChromaTOF (LECO). Peak areas were integrated using Skyline (23.0, MacCoss Lab Software), before quantification against external calibration curves and curation (2022b, MathWorks). Matrix effects, carry over, noise levels, and precision were evaluated using corresponding quality control samples. This method specifically targets SCFAs, vital in maintaining the gut-liver axis balance by supporting gut barrier integrity and regulating metabolism.

### Determination of Cell Viability Via XTT Assays

For viability experiments, HepG2 cells were seeded into collagen-coated 96-well plates (40 × 10^4^ cells/well), in FluoroBrite DMEM (Gibco), a phenol red-free medium to prevent nonspecific colorimetric interference, supplemented with 5% glutamine (GlutaMax, Gibco) and 10% FCS. Culture dishes were incubated overnight (5% CO_2_, 37°C, 95% relative humidity). Cell viability was assessed using sodium 3'-(1-(phenylaminocarbonyl)-3,4-tetrazolium)-bis(4-methoxy-6-nitro) benzene sulfonic acid hydrate (XTT) assay (Abcam). XTT reagent was added to each well and incubated for 2 hours. Subsequently, cell metabolic activity was monitored hourly over a 4-hour period. Kinetic reads of defined time points were performed at 450 to 620 nm using a microplate Sunrise-Basic reader (TECAN). This assay evaluates mitochondrial dehydrogenase activity via reduction of XTT (sodium 3'-(1-(phenylaminocarbonyl)-3,4-tetrazolium)-bis(4-methoxy-6-nitro) benzene sulfonic acid hydrate) to a water-soluble orange formazan. Formazan formation correlates with the number of metabolically active cells, driven by nicotinamide adenine dinucleotide phosphate, reduced form (NADH)-dependent electron transfer across the plasma membrane. The water-soluble formazan dissolves into the medium, allowing real-time kinetic measurements (according to the manufacturer’s instructions provided in the datasheet of the kit).^42^ HepG2 viability was analyzed after exposure to 6-hour infected-Caco-2 conditioned medium or selected metabolites, with rigorous controls. Cell viability was calculated as follows:

$cell\;viability\;\%=\frac{OD\;treated\;sample-OD\;control}{OD\;untreated\;sample}*100$.

### Determination of HepG2 Cell Viability After Stimulation With Caco-2 Conditioned Medium

To perform HepG2 cell viability after exposure to Caco-2 conditioned medium, Caco-2 cells were seeded in 24-well plate and infected with *A* *baumannii* or *K* *pneumoniae* strains for 6 hours. Conditioned media from uninfected Caco-2 cells and bacteria grown without host cells, incubated for 6 hours, served as rigorous control groups to ensure the robustness of the results. Supernatants were collected, centrifuged at 4°C (20.817 × g for 10 minutes), filter sterilized (0.22 μm) and stored at −80°C prior to use. Sterility was confirmed by incubating 100 μL of each supernatant on CBA plates for 24 hours. HepG2 cells, pre-seeded in a 96-well plate, were exposed to conditioned media at 5 different volumes, such as 100 μL, 50 μL, 25 μL, 10 μL, and 5 μL. Each well was supplemented with FluoroBrite DMEM (phenol red–free, FCS-free) to achieve a final volume of 100 μL. Exposure durations included 2 hours, 3 hours, and 4 hours. HepG2 cell viability was determined via XTT assay as described above.

### Metabolite Preparation for Evaluating HepG2 Cell Viability After Exposure

To assess HepG2 cell viability after exposure to selected metabolites identified previously via metabolomics of Caco-2 infection supernatants and controls (uninfected Caco-2 and bacteria-only cultures), the following compounds were tested: α-ketoglutarate, indole-3 acetic acid, p-coumaric acid, uridine, desthiobiotin, N-acetylglutamine (all Sigma-Aldrich); ß-pinene (Roth); and N8-acetylspermidine (Chemos). Stock solutions were freshly prepared in DMSO (Roth), at the following concentrations: α-ketoglutarate, uridine: 10 mg/mL; indole-3 acetic acid, p-coumaric acid: 15 mg/mL; desthiobiotin, ß-pinene: 20 mg/mL; N8-acetylspermidine, N-acetylglutamine: 30 mg/mL. Stocks were diluted in FluoroBrite DMEM (phenol red–free, FCS-free) to final concentrations of 50, 100, 250, and 500 μg/mL. HepG2 cells were exposed for 2, 3, and 4 hours, and viability was measured via XTT assay. Two compound mixtures were also tested at identical concentrations and time points: (1) α-ketoglutarate, indole-3 acetic acid, p-coumaric acid, uridine (from *A* *baumannii* experiments); and (2) desthiobiotin, ß-pinene, N8-acetylspermidine, N-acetylglutamine (from *K* *pneumoniae* experiments), each containing equal amounts of the respective metabolites. DMSO-only controls matched solvent concentrations to ensure that observed effects on HepG2 cells were metabolite-specific.

### Patients and Healthy Volunteers

#### Frankfurt cohort

To validate in vitro findings, clinically unannotated serum samples from patients with ACLF were retrospectively analyzed within the ongoing prospective longitudinal study “Characterization and Pathogenesis of ACLF” (ACLF-I, NCT04975490). Adult patients with liver cirrhosis admitted to the Department of Internal Medicine 1 at University Hospital Frankfurt between 2021 and 2024 were enrolled. Cirrhosis was diagnosed based on liver biopsy or a combination of clinical, laboratory, imaging, and endoscopic findings. ACLF was defined according to CANONIC study criteria,^1^ and organ failure assessed using the CLIF-C OF score.^43^ For metabolomics, a subgroup of patients with ACLF with alcohol-related liver cirrhosis (N = 20) was selected. Alcohol-related etiology was defined by a reported daily alcohol intake exceeding 20 g/day. Healthy controls (N = 11) were included using samples obtained from the DRK Blood Donation Service Baden-Württemberg/Hessen (Table 6). Ethical approval was granted by the Institutional Ethics Committee of University Hospital Frankfurt (Ref. 20-653 and 329-10), and written informed consent was obtained from all participants in accordance with the Declaration of Helsinki.

#### Bonn/Münster cohort

Serum samples from 32 patients with acutely decompensated liver cirrhosis as part of the NEPTUN study (NCT03628807) collected between 2014 and 2018 at the Department of Internal Medicine I, University Clinic Bonn (Germany) were used for validation. During TIPS insertion, blood samples were collected from the portal (PV), hepatic (LV), and cubital veins (CV) of all patients for laboratory serum and metabolic profiling. Disease severity in these patients was assessed by MELD score,^44^ Child-Pugh score, CLIF acute decompensation (CLIF C AD) and ACLF (CLIF C ACLF) scores,^43^ respectively (Table 7). The study was approved by the local ethics committee of the University of Bonn (029/13 and 203/13), and all patients signed an informed written consent following the Helsinki Declaration.^45^

### Blood Sampling and Data Collection

Clinical data, laboratory data, and serum samples were obtained at baseline. Routine laboratory diagnostics included liver function tests, differential WBC count, CRP, AST, alanine transaminase (ALT), INR, drinking and smoking behavior, gastrointestinal bleeding, ascites, therapeutic paracentesis, albumin treatments, diabetes and diabetes treatment, TIPS, hepatitis/viral infections, renal failure, respiratory failure, circulatory failure, and data on bacterial and/or fungal infection development.

### Processing of Blood Samples and Serum Isolation

Serum samples (9 mL) from patients with ACLF and healthy controls centrifuged at 1400 × g for 10 min at 4°C. The supernatant (serum) was aliquoted and stored at −80°C until further use.

### Metabolomics Analysis of Patient Sera

#### Frankfurt cohort

Metabolite analysis was performed using liquid chromatography-high resolution mass spectrometry (LC-HRMS). Briefly, 10 μL of serum was mixed with 20 μL of methanol, 10 μL of 800 ng/mL uridine-13C5 in methanol and 70 μL of acetonitrile, then incubated at −20°C for 2 hours. After centrifugation (4°C at 18.000 × g for 10 minutes), 80 μL of the supernatant was evaporated under nitrogen at 45°C and reconstituted in 80 μL of 50% acetonitrile. Samples were randomized and included pooled serum from 10 random samples and human ethylene diamine tetra-acetic acid (EDTA) plasma replicates as quality controls. Reference standards SRM 1950 NIST (Thermo Fisher Scientific) were diluted in methanol and extracted similar to the samples with 10 μL of DPBS instead of serum, and 20 μL of the respective standard solution instead of methanol. LC-HRMS was performed on an Orbitrap Exploris 480 with a Vanquish Horizon UHPLC system (both Thermo Fisher Scientific), operating both in positive and negative ion mode. The separation was achieved using a SeQuant ZIC-HILIC column (3.5 μm, 100 mm × 2.1 mm I.D., Merck) with a same type of guard column and an inline filter. Data were acquired using XCalibur v4.4 software and evaluated using TraceFinder software v5.1 (both Thermo Fisher Scientific).

#### Bonn/Münster cohort

Metabolite extraction was performed twice for further LC/HRMS analyses with 2 chromatographic columns (ie, HILIC and C18) from 50 μL of plasma using methanol-assisted protein precipitation, as previously described.^46^ Briefly, a volume of 200 μL of methanol containing internal standards at 3.75 μg/mL (dimetridazole, 2-amino-3-(3-hydroxy-5-methyl-isoxazol-4-yl) propanoic acid [AMPA], 2-methyl-4-chlorophenoxyacetic acid [MCPA], and Dinoseb [Sigma-Aldrich]) was added to the 50 μL of plasma. The resulting samples were then left on ice for 90 minutes until complete protein precipitation. After a centrifugation step at 20,000 g for 15 minutes at 4°C, supernatants were collected and dried under a nitrogen stream using a TurboVap instrument (Thermo Fisher Scientific) and stored at −80°C until analysis. Prior to LC-MS analysis, dried extracts were resuspended in 150 μL of ammonium carbonate 10 mM pH10.5 + external standards solution (ESS∗)/Acetonitrile (40:60, v/v) for ZIC-pHILIC analysis or H2O/Acetonitrile (95:5, v/v), containing 0.1% formic acid and ESS∗ for C18 analysis. After reconstitution, the tubes were vortexed and incubated in an ultrasonic bath for 5 minutes and then centrifuged at 20,000 g for 15 minutes at 4°C. Supernatant was transferred into 0.2 mL vials. A quality control (QC) sample was obtained by pooling 20 μL of each sample preparation. QC samples were injected every 10 samples to evaluate the signal variations of any metabolite.

ESS∗, a mixture of 9 authentic chemical standards covering the mass range of interest (13C-glucose, 15N-aspartate, ethylmalonic acid, amiloride, prednisone, metformin, atropine sulfate, colchicine, imipramine) was added to all samples to check the consistency of analytical results in terms of signal and retention time stability during the experiments.

Untargeted metabolomics experiments were performed by LC-HRMS, using a combination of 2 complementary chromatographic methods^46^ consisting of reversed-phase chromatography (C18 chromatographic column) and hydrophilic interaction chromatography (HILIC) for the analysis of hydrophobic and polar metabolites, respectively. LC-HRMS experiments were performed by using an Ultimate 3000 chromatographic system (Thermo Fisher Scientific) coupled to an Exactive mass spectrometer from Thermo Fisher Scientific fitted with an electrospray (ESI) source and operating in the positive and negative ion modes for metabolite separations on C18 and HILIC columns (see below), respectively. Chromatographic conditions were exactly those previously described by our group,^46^^,^^47^ whereas the mass spectrometer was operated with capillary voltage at −3 kV in the negative ionization mode and 3 kV in the positive ionization mode and the capillary temperature set at 280°C. The sheath gas pressure and the auxiliary gas pressure were set at 60 and 20 arbitrary units with nitrogen gas, respectively. The detection was achieved from m/z 70 to 1000 at 50,000 resolution in both ionization modes. Data acquisition was performed in a single batch, in a randomized order, with quality control samples injected regularly. Data processing analyses were performed using the Workflow4Metabolomics (W4M) platform.^47^ Metabolite features were first annotated according to accurate measured masses and chromatographic retention times by using our spectral database^47^^,^^48^ and filtered by peak correlation, repeatability, and biological-to-blank peak ratios. Metabolite identification was further confirmed using MS/MS data collected under non-resonant collision-induced dissociation conditions using higher-energy C-trap dissociation (HCD) using a Q-Exactive mass spectrometer from Thermo Fisher Scientific. MS/MS data were further matched both manually using Xcalibur constructor software (Thermo Fisher Scientific) and automatically using the MS-DIAL software to the spectra included in our in-house spectral database, as previously described.^48^ To be identified, metabolites had to match at least 2 orthogonal criteria (ie, accurate measured mass, retention time, MS/MS spectrum) to those of an authentic chemical standard analyzed under the same analytical conditions, as proposed by the Metabolomics Standards Initiative.^46^

### Statistics and Data Analysis

All experiments were performed in triplicates, at least 3 times, independently. Values are means ± standard deviation (SD). Statistical analyses included χ^2^ (for comparison of categorical variables), 1-way analysis of variance (ANOVA) (to assess effect of a single independent variable) and a 2-way ANOVA (to evaluate effects of 2 independent variables), using GraphPad Prism V8 (GraphPad). A value of *P* < .05 was considered statistically significant. For immunofluorescence and microscopy, representative pictures from at least 25 high-power fields are depicted.

### Ethics

This study was approved by the Institutional Ethics Committee University Hospital Frankfurt am Main (Germany) with reference number 20-653 for ACLF-I cohort patients’ sera, and reference number 329-10 for the DRK Blood Donation Service Baden-Württemberg/Hessen (Frankfurt am Main, Germany) patients’ sera. The blood collection and analysis in the validation cohort were approved by the local ethics committee of the University of Bonn (029/13 and 203/13). Written consent was obtained from all participants in accordance with the Declaration of Helsinki.
